# Analysis on the Mechanical Behavior of Flexible Screens

**DOI:** 10.3390/ma15082829

**Published:** 2022-04-12

**Authors:** Lirui Niu, Jun Ding, Wei Liu

**Affiliations:** 1 School of Mechanical Engineering, Southwest Petroleum University, Chengdu 610500, China; lingdensity@gmail.com; 2 Center for Gravitational Wave Experiment, Institute of Mechanics, Chinese Academy of Sciences, Beijing 100190, China

**Keywords:** OLED flexible screen, bending radius, thickness of the shape, water-drop-shaped

## Abstract

Recently, flexible organic light-emitting devices (OLEDs) have become more and more popular. However, the force distribution and deformation are very complex during the bending process, and it is difficult to analyze the stress and strain by theoretical analysis and direct experiment. In this paper, finite element analysis of the bending model for the flexible screen was performed. For common U-shaped bending, it was shown that the maximum Mises stress increases rapidly as the bending radius decreases, and the redistribution of the tensile zone and the compression zone should be the key to the layer material selection. The results were verified by an imaging experiment. Further, a water-drop-shaped bending mode was analyzed to reduce the risk of structure failure.

## 1. Introduction

In recent years, flexible organic light-emitting devices (OLEDs) have played an important role in flexible displays, smart wear and other fields. Compared with traditional liquid crystal display (LCD) screens, flexible OLEDs have excellent characteristics, such as self-illumination, wide viewing angle, light weight, low energy consumption and flexibility. With the increasingly wide use of OLEDs in display products, the structural optimization and stability of the OLED flexible screen have become important metrics to meet the considerations of portability and economy.

The OLED display module is a composite stacked structure composed of different thin optical films, and each layer is bonded by an optical clear adhesive (OCA) material with both hyperelasticity and viscoelasticity. During the bending process, the small bending radius causes the device to be overstressed, meanwhile, the inconsistent deformation between various thin films leads to the viscous flow of the OCA, which tends to make the devices peel off and cause permanent damage to the screen. Because the force distribution and deformation are very complex during the bending process, it is difficult to analyze the stress and strain by theoretical analyses. It is also difficult to observe and measure the stress and strain distributions directly during the whole bending process in experiments. Therefore, the finite element method which can obtain the stress and strain results was employed to further reveal the damage mechanism and adhesive layer peeling of the flexible OLED screen.

Based on the level of basic materials, a new type of hole-transport layer for host material was developed by using a new method (ball milling process) and green halogen-free solvent [1,2], which not only improved the photoelectric response level of the optical monomer thin film device, but also showed good stability under continuous stress. Krucaite et al. [3] studied the use of the carbazole rings or substituted carbazole-containing compounds, so that the prepared single optical thin film device had better optical and mechanical properties. The performance optimization of OLED flexible screen monomer devices was studied [4,5,6,7,8,9,10]. It is concluded that the improvement of the bending performance for the single component is beneficial to the performance optimization of the overall module. However, the stress situation of the stack is quite different when it is bonded with OCA. Based on the constitutive equation and superposition of OCA, some scholars [11,12,13,14,15,16,17,18,19,20,21,22,23,24,25,26] have also carried out extensive research using finite element analysis, and the stress neutral layer phenomenon is proposed. However, there is a certain gap between the stacking structure and the actual flexible screen module, for example, the influence of the material parameters of key laminated OCA and colorless polyimide (CPI), and the bending radius and bending angle of the flexible screen module on the structure is not well understood.

In this study, finite element software ABAQUS (2020, Dassault Systemes Simulia Corp., Johnston, RI, USA) was introduced to establish the real stacking model of the flexible screen module. The influence of the bending radius, OCA thickness-shape and bending mode (including U-shaped and water-drop-shaped) on the mechanical behavior of the OLED flexible screen are discussed. To verify the results of the analysis, an imaging experiment was performed.

## 2. Finite Element Model

**Figure 1:**
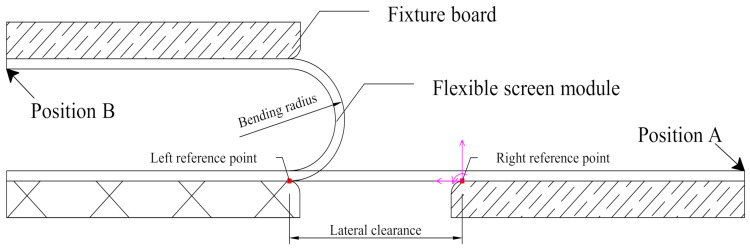
Geometric structure model of U-shaped bending mode. The left reference point was fixed, and the bending was performed by moving the fixture board from position A to position B within the bending time *t* seconds. The lateral gap was *πR* since the bending angle was *π* and the bending radius was *R*.

In this paper, the finite element model was established using ABAQUS to simulate the mechanical behavior for the flexible screen. As shown in Figure 1, the screen module and a fixture board were glued together, and the bending of the screen was driven by the movement of the fixture board. For a common U-shaped bending mode, the bending angle was set to be *π*, the bending radius was *R*, the initial lateral gap was *πR* and the bending was completed in *t* seconds.

A typical OLED flexible screen is stacked with a BMS (support), CPI, BP (back-plane), OCA, POL (polarizer), TW (touch-writing), PD (prevent-damage) and an OLED light-emitting surface. Among them, OCA is mainly made of acrylic material. Foam glue is often used in the test, the core light-emitting layer of OLED is complex in composition, mostly composed of minerals, organics, metal oxides, etc., and the remaining film layers are mostly optical engineering films composed of PET, TAC, and PVA. The stacking order and the corresponding thickness from the top to the bottom of the flexible screen module are given in Table 1. The fixture board was regarded as a rigid body in this simulation as it is not the focus of the analysis. The glue layer binds the screen module and the fixture board. Although the OLED, PD and TW layers are all composed of multiple layered materials, they can be taken as a composite material layer based on the composite material mechanical laminate theory, which would simplify the finite element model.

## 3. Constitutive Equations and Material Parameters

The flexible screen module was composed of multilayered films with different mechanical properties. The film layers were bonded by OCA, and the deformation of each layer during the folding process was co-ordinated which plays a key role in the structure of the flexible screen module.

As discussed in [27], OCA has hyperelastic and viscoelastic properties. In order to describe these OCA attributes reasonably, the Ogden constitutive equation and Prony constitutive equation were introduced [27]. The hyperelastic Ogden constitutive equation is described as follows:

(1) $$U\;=\;\underset{i\;=\;1}{\overset{N}{\sum }}\frac{2\mu_{i}}{\alpha_{i}^{2}}({\bar{\lambda }}_{1}^{\alpha_{i}}\;+\;{\bar{\lambda }}_{2}^{\alpha_{i}}\;+\;{\bar{\lambda }}_{3}^{\alpha_{i}}\;-\;3)\;+\;\overset{N}{\underset{i\;=\;1}{\sum }}\frac{1}{D_{i}}{\left(J\;-\;1\right)}^{2i}$$

where ${\bar{\lambda }}_{i}$ is the principal tensile deviator, ${\bar{\lambda }}_{i}\;=\;J^{\;-\;\frac{1}{3}}\lambda_{i}$, $J\;=\;{\left(\lambda_{1}\lambda_{2}\lambda_{3}\right)}^{\frac{1}{2}}$ is the ratio of total volume, $\lambda_{i}$ is the principal tensile, $\alpha_{i}$ and $\mu_{i}$ are strain hardening exponent and shear modulus, respectively, $D_{i}$ is the compressible parameter and N is the order. Due to uniaxial tensile deformation and incompressibility, the sum of the latter term in Equation (1) is set to be zero. Thus, the stress is given by the following equation:

(2) $$\sigma_{UT}=\frac{\partial U}{\partial \lambda_{{}_{UT}}}\;=\;\underset{i\;=\;1}{\overset{N}{\sum }}\frac{2\mu_{i}}{\alpha_{i}}\cdot J^{\;-\;\frac{\alpha_{i}}{3}}(\lambda_{{}_{UT}}^{{}^{\alpha_{i}\;-\;1}}\;-\;\lambda_{{}_{UT}}^{{}^{\;-\;\frac{\alpha_{i}\;+\;2}{2}}})$$

The viscoelastic behavior of the time domain is determined by the time-dependent shear modulus $G_{R}(t)$ and its normalized Prony equation is shown in Equation (3) [28]:

(3) $$\frac{G_{R}(t)}{G_{0}}\;=\;1\;-\;\overset{N}{\underset{i\;=\;1}{\sum }}{\bar{g}}_{i}^{P}(1\;-\;e^{\;-\;\frac{t}{\tau_{i}}})$$

where $G_{0}$ is the instantaneous shear modulus, ${\bar{g}}_{i}^{P}$ is the expression coefficient and $\tau_{i}$ is the state variable to control the stress relaxation.

**Figure 2:**
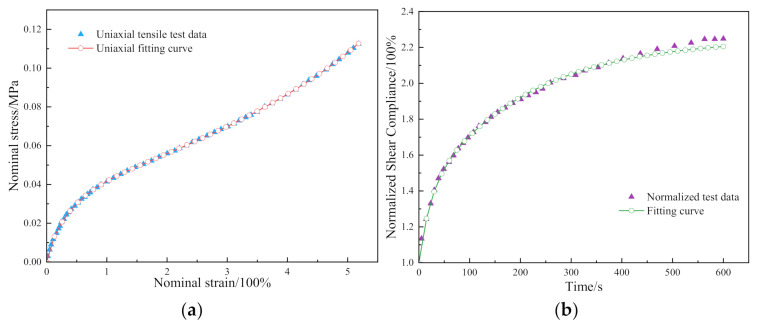
(**a**) The fitting result of the OCA for the Ogden model; (**b**) the fitting result of the OCA for the Prony model.

The material parameters of the OCA used in the Ogden and Prony equations are shown in Table 2 and Table 3, respectively. The corresponding fitting curves were obtained from Figure 2 which were in good agreement with the experimental results.

Further, the elastic modulus of the OLED, PD and TW layers were estimated according to the composite material mechanical laminate theory using the following formula [29]:

(4) $$E\;=\;\frac{\sum E_{i}\cdot t_{i}}{\sum t_{i}}\;=\;\frac{E_{1}\cdot t_{1}\cdot n_{1}\;+\;E_{2}\cdot t_{2}\cdot n_{2}\;+\;\cdots \;+\;E_{n}\cdot t_{n}\cdot n_{n}}{t_{1}\cdot n_{1}\;+\;t_{2}\cdot n_{2}\;+\;\cdots \;+\;t_{n}\cdot n_{n}}$$

where $E_{i}$, $n_{i}$ and $t_{i}$ are the elastic modulus, number of layers and thickness of the i-th material, respectively.

A plastic linear strengthening model was employed for the CPI. The corresponding stress and strain parameters are shown in Table 4. For other layers in the stack model, the relevant material parameters are listed in Table 5.

## 4. Results and Discussion

### 4.1. Influence of the Bending Radius

At present, screen manufacturers and consumers have imposed demanding requirements for the thickness of flexible screens. Thus, the bending radius plays a key role in the foldability of the flexible screen components. In this paper, the bending radii were set to be 3 mm, 2.5 mm, 2 mm, 1.5 mm and 1 mm, respectively, and the bending time *t* = 18 s was used for a 180-degree folding simulation.

**Figure 3:**
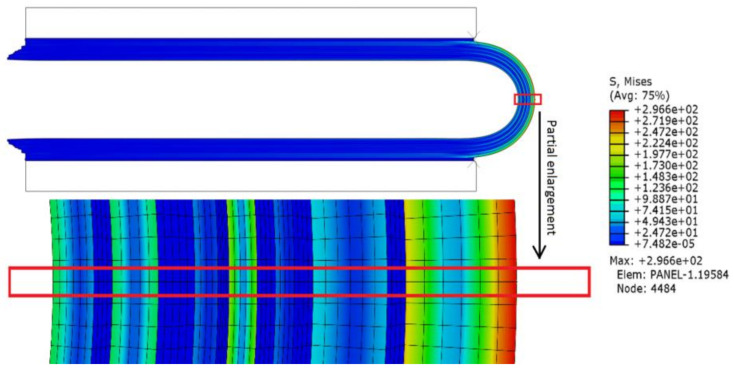
Mises stress distribution after downward bending.

Figure 3 shows the Mises stress distribution in the case of *R* = 2 mm. As the bending radius decreases: the curvature radius is inversely proportional to the normal bending stress under the differential geometry; the Mises stress of each film layer increases non-linearly; and the maximum value is located at the outermost position of the symmetrical central axis (hereinafter referred to as the central axis, marked by the red box in Figure 3 after folding). Therefore, the decrease in the bending radius makes the risk of film damage gradually increase.

**Figure 4:**
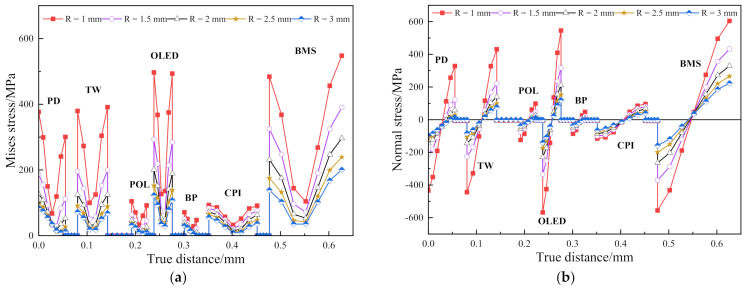
(**a**) Mises stress distribution along the central axis at different bending radii; (**b**) contrast curve of normal bending stress of the central axis at different bending radii.

If the slight displacement of the central axis is ignored, the Mises stress distribution along the central axis for each bending radius can be obtained from Figure 4a in which the inner end point of the axis is taken as the origin. It can be seen that, in the film layers other than the OCA adhesive layer, the Mises stress first drops and then rises while the stress of the OCA layer approaches 0 and the outermost edge of the BMS layer stress reaches the maximum. As the bending radius decreases, the Mises stress of each layer along the central axis gradually increases non-linearly. Therefore, the small bending radius requires high film material strength to support the entire bending process.

**Figure 5:**
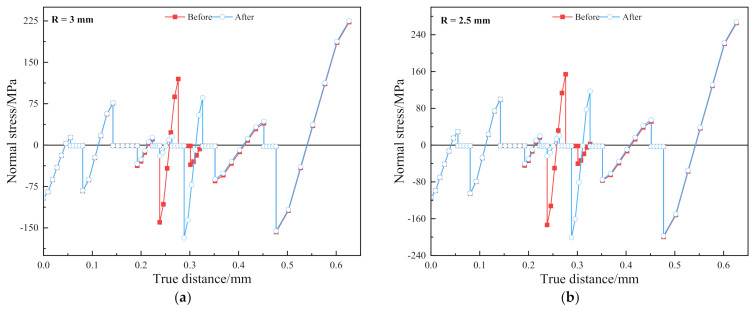
(**a**) Contrast curve of normal bending stress along the central axis before and after the interchange of the OLED and BP layers under *R* = 3 mm; (**b**) contrast curve of normal bending stress along the central axis before and after the interchange at *R* = 2.5 mm.

Figure 4b shows the normal bending stress along the central axis. Each film layer appears to be compressed and then stretched. The position of the local neutral layer is the position where the normal stress is 0. Since the normal bending stress near the neutral layer is the lowest, it is speculated that if the position of the OLED and BP layers are interchanged, the risk of damage to the OLED layer is reduced. Figure 5a,b show the comparison graphs of normal bending stress after the OLED and BP layers are interchanged at *R* = 3 mm and 2.5 mm, respectively.

It can be seen from Figure 5 that for *R* = 2.5 mm, the maximum tensile stress is reduced by 23.99% and the maximum compressive stress is increased by 15.82%. When *R* = 3 mm, the tensile stress is reduced by 27.54% and the maximum compressive stress is increased by 20.22%. This indicates that after the interchange, the demand for compressive load-bearing increases while the demand for tensile load-bearing decreases. After the OLED and BP layers are interchanged, the risk of strength failure increases. Therefore, for the composite stack model, a single local optimization may not have a positive effect on the overall structure.

**Figure 6:**
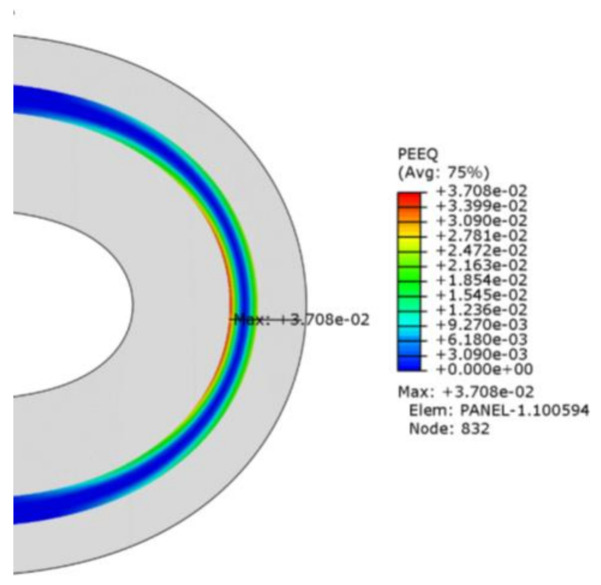
Distribution of equivalent plastic strain after bending with *R* = 1 mm.

Due to the plastic nature of CPI, the CPI layer has not yet undergone plastic deformation with *R* = 3 mm. When the bending radius is reduced from 2.5 mm to 1 mm, the maximum equivalent plastic strain is 6.234 × 10^−4^, 6.108 × 10^−3^, 1.498 × 10^−2^ and 3.708 × 10^−2^. Figure 6 shows the maximum equivalent plastic strain area with *R* = 1 mm.

When the bending radius is 2.5 mm, the plastic strain occurs first from the inside of the CPI layer, that is, the plastic strain occurs first in the compressed area. The plastic strain in the outer tension area does not occur until the bending radius is 2 mm. When the bending radius is 1.5 mm, the plastic strain occurs in the outer tension area. As the bending radius continues to decrease, the inner compression and outer tension areas expand. Because the compression zone inside the CPI first enters the plastic phase, if the CPI lamination stress reaches or exceeds the critical stress of buckling, it will cause the film to buckle, which is an unstable form of film failure. The buckling of two types of films with different physical forms, such as wrinkles and delamination, is mainly caused by compression effects. This is one of the reasons why the central axis position of the flexible screen module is raised inward after multiple bending. In order to prevent irreversible damage to the flexible screen, if the bending radius changes during the product design, the stacking layer sequence and material selection should be adjusted accordingly, and the product use process should avoid external factors that cause the bending area to be too small. In Section 4.3, water-drop shaped folding was discussed to avoid the restriction of the bending area.

### 4.2. Influence of the OCA Profile

**Figure 7:**
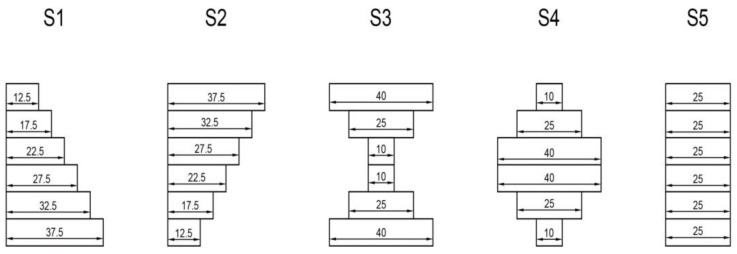
Five OCA layer profiles (units shown in μm).

Since the film materials and the stacking sequences of the flexible screens are tending to become well-established, the OCA layer has become the preferred optimization object under the current engineering background. In order to study the influence of the OCA layer profile on the light-emitting layer of the OLED during the folding process, five OCA profiles, shown in Figure 7, were carried out for the finite element analysis when the total thickness of the OCA layers remained unchanged.

The stacking sequence is shown in Table 1. The profiles of the OCA adhesive layer in Figure 7 corresponds to the thickness of the OCA layers in Table 1: the 2nd, 4th, 6th, 8th, 10th and 12th layers. The seven functional layers and six adhesive OCA layers were alternately stacked, the bending radius was set to be *R* = 3 mm and the bending time was *t* = 18 s.

**Figure 8:**
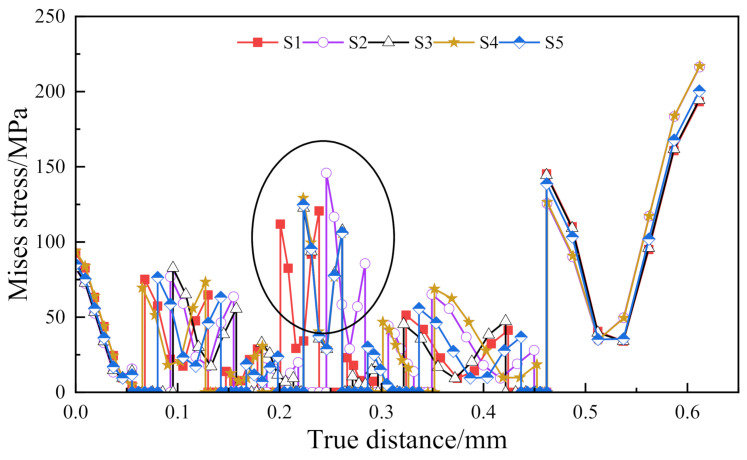
Mises stress distribution under the different OCA profiles. The Mises stress of the circled area is the stress of the OLED light-emitting layer.

The Mises stress distribution along the central axis under the five OCA profiles is shown in Figure 8. The maximum Mises stress is at the outermost position of the structure, and the selected part in the figure is the Mises stress of the OLED light-emitting layer.

It can be seen that the profiles S3, S4 and S5 share similar effects on the OLED light-emitting layer; the maximum difference ratios of S3/S5 and S4/S5 are 1.27% and 3.61%, respectively. On the other hand, compared with S5, the maximum stress of the OLED layer caused by profiles S1 and S2 increased by 13.53% and 24.04%, respectively. Further, the increase in the stress in the tension or compression zone on one side of the layer would correspondingly decrease the stress in the compression or tension zone on the other side. In other words, the profile of the OCA layer can be used to optimize the dominant bending tension or compression of the OLED layer.

**Figure 9:**
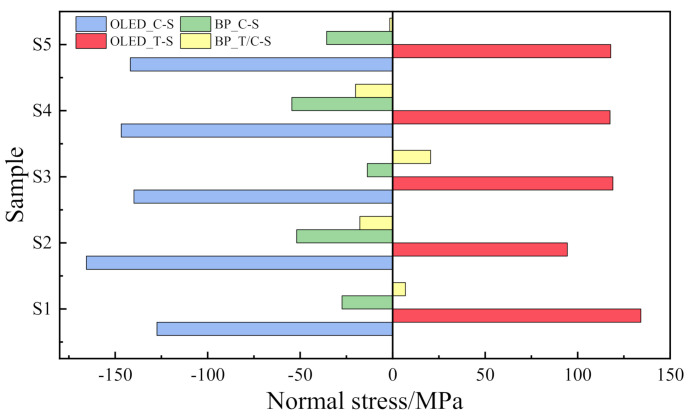
Contrast of normal stress in bending of the central axis with different thickness shapes.

In order to investigate the influence of the OCA profiles on the normal bending stress of the OLED and BP layers, a comparison diagram of the normal bending stress along the central axis under the five OCA profiles is shown in Figure 9. The abbreviation C-S means compression stress, and T-S means tensile stress and T/C-S means tensile or compression stress.

In the OLED, areas S3, S4 and S5 share a similar stress distribution while S1 and S2 are different, which proves that the adhesive layer profile will change the tension or pressure dominance of the OLED light-emitting layer. On the other hand, the BP layer is completely dominated by compressive stress under S2, S4 and S5, while S1 and S3 create tensile stress. Therefore, to reduce the probability of yield failure, normal stress of the flexible screen module can be modulated according to the material characteristics of the laminated stack structure.

**Figure 10:**
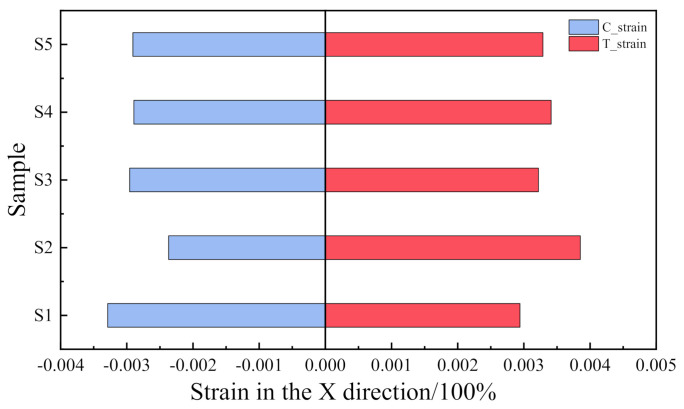
Strain comparison of OLED layer in the axial x direction with different profiles.

The impact of the OCA layer on the OLED layer should be investigated. Because of its superelasticity, the OCA has an obvious strain response during the folding process. Considering its impact on the OLED layer, the strain of the OLED layer in the same direction as the central axis was used to draw the sample-strain diagram in Figure 10. The abbreviation C-strain in Figure 10 means compression strain and T-strain means tensile strain.

It can be seen from Figure 10 that the maximum increment ratio of S1/S5 is 13.07%, the maximum increment ratio of S2/S5 is 18.56%, the absolute strain increment ratios of S1/S5 and S2/S5 are 1.0053% and 1.0042%, respectively. Therefore, the change of the profile does not have a critical effect on the absolute difference of the strain along the x direction of the OLED layer, but it will offset the neutral layer to the left or right, which is still dominated by changing the tensile strain or compressive strain of the OLED layer.

### 4.3. Influence of the Water-Drop Folding Shape

**Figure 11:**
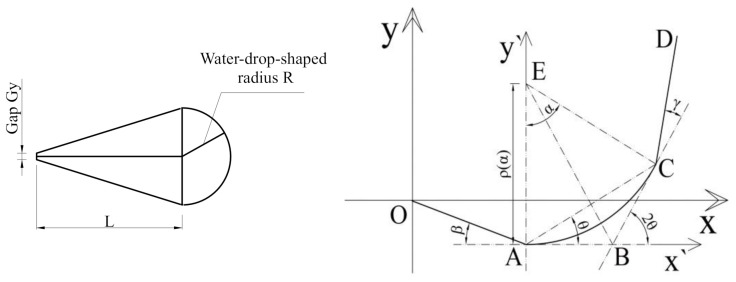
(**a**) Simplified water-drop geometry; (**b**) geometric relationship of the water-drop-shaped.

At present, U-shaped full-path folding is adopted by most producers and companies. However, different folding forms are employed gradually in order to reduce the bending internal stress, among which, the water-drop-shaped folding is introduced into the flexible electronics field due to its superior load-bearing capacity of the structure and increased ability to reduce the gap between the two folded screens [30,31]. The water-drop shape means that the shape of the folded flexible screens is like a water-drop, which is shown in Figure 11a. Therefore, the shape of the water-drop in the final bending state was used as the design blueprint. The design bending radius was *R*, the distance between the center of the water-drop semicircle and the tip of the water-drop was *L* and the gap between the two screens was Gy. The folding process was controlled by the parameters *R*, *L*, Gy and the motion. In order to ensure the evenly distribution of the bending stress in the bending area of the flexible screen, the bending area was in a symmetrical bending state at all times. At the same time, the servo control equation was used to control the water-drop-shaped bending process. The geometric relationship of the water-drop-shaped bending process is shown in Figure 11b.

In order to derive the water-drop-shaped servo control curve equation, the co-ordinate system x`Ay` was first obtained as shown in Figure 11b. Since the tangent angles are equal at two points of symmetry on the arc $\overset{⏜}{\mathrm{AC}}$ at any time, the following equations can be derived from geometric relations:

(5) $$\left\{\begin{array}{c} \alpha \;=\;2\theta \\ \bar{AB}=\;\bar{BC}\\ \rho (\alpha )\cdot \alpha \;=\;\pi R\\ \bar{AB}\;=\;\tan\frac{\alpha }{2}\cdot \rho (\alpha )\\ \cos∠ABC\;=\;\frac{{\bar{AB}}^{2}\;+\;{\bar{BC}}^{2}\;-\;{\bar{AC}}^{2}}{2\bar{AB\cdot }\bar{BC}} \end{array}\right.$$

The trajectory equation of point C is expressed as follows with the polar co-ordinate in the co-ordinate system x`Ay` by solving Equation (5):

(6) $$r(\theta )\;=\;\pi R\cdot \frac{\sin\theta }{\theta };\;\;\;\;\;\theta \;\in \;(0,\frac{\pi }{2}]$$

The water-drop-shaped bending path is described by the trajectory equation of point D. In the co-ordinate system xOy, the additional geometric parameters of the water-drop shape can be obtained from Figure 11a. Since the servo control equation is a function of time *t* and the total control time is set to be *T*, to ensure a uniform rotation angle per unit time, the following expressions are obtained by combining geometric relationship:

(7) $$\left\{\begin{array}{c} \bar{OA}\;=\;\bar{CD}\;=\;\sqrt{\frac{1}{4}{\left(2R\;-\;G_{y}\right)}^{2}\;+\;L^{2}}\\ \theta \;=\;\frac{\pi t}{2T};\;t\in [0,T]\\ \beta \;=\;\gamma =\frac{t}{T}\cdot \arctan\frac{2R\;-\;G_{y}}{2L};\;t\;\in \;[0,T] \end{array}\right.$$

Thus, the drop-shaped bending servo control curve (Equation (8)) can be derived to obtain the servo co-ordinates of point D at any time *t* within the total time *T* by converting Equation (6) under the co-ordinate system x`Ay` into xOy co-ordinate and taking Equation (7) into account:

(8) $$\left\{\begin{array}{c} \begin{array}{l} l_{x}(t)\;=\;\frac{R}{t}\cdot (\pi t\;-\;T\cdot \sin\frac{\pi t}{T})\;+\;\bar{OA}\cdot (2\;-\;\cos(\frac{t}{T}\cdot \\ \left(\pi +\arctan\frac{2R\;-\;G_{y}}{2L}))\;-\;\cos(\frac{t}{T}\cdot \arctan\frac{2R\;-\;G_{y}}{2L})\right) \end{array}\\ \begin{array}{l} l_{y}(t)\;=\;\frac{R}{t}\cdot (T\;-\;T\cdot \cos\frac{\pi t}{T})\;+\;\bar{OA}\cdot (\sin(\frac{t}{T}\cdot \\ \left(\pi \;+\;\arctan\frac{2R\;-\;G_{y}}{2L}))\;-\;\sin(\frac{t}{T}\cdot \arctan\frac{2R\;-\;G_{y}}{2L})\right) \end{array} \end{array}\right.;\;t\;\in \;(0,T]$$

**Figure 12:**
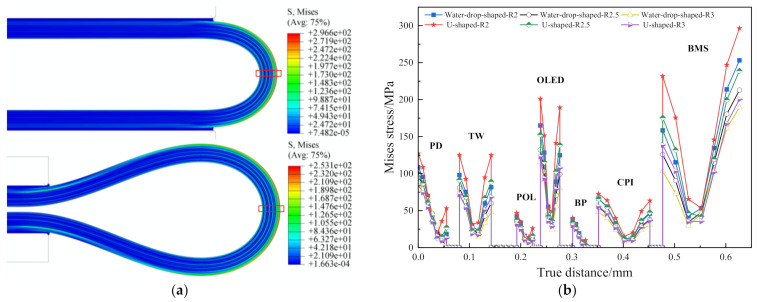
(**a**) Mises stress distribution for two types; (**b**) Mises stress comparison curve.

Thus, the U-shaped bending and the water-drop-shaped bending were simulated under *L* = 3 *R*, Gy = 0.2 mm and *R* was set to 3 mm, 2.5 mm, 2 mm, respectively; and the results were shown in Figure 12. The Mises stress distribution for two types under *R* = 2 mm is shown in Figure 12a.

It can be seen from Figure 12a that the most dangerous position of the flexible screen after the water-drop-shaped and U-shaped folding, that is, the maximum Mises stress, is marked by the red box at the position of the central axis of the bending. The Mises stress diagram of six types of samples along the bending axis is shown in Figure 12b. It can be seen from Figure 12b that for each functional layer of the flexible screen, under the different bending radii, the water-drop-shaped bending results in a smaller stress response than the U-shaped bending. The smaller the bending radius, the more obvious the water-drop folding is at reducing the maximum Mises stress since the light-emitting layer of the OLED layer, the flexible substrate of the CPI layer and the BMS layer are the core concern about failure of the structure. Table 6 shows the maximum stress response for the three types of core membranes under the two folding types. The increment ratio refers to the ratio of the difference between the maximum stress of the U shape and the maximum stress of the water-drop shape to the maximum stress of the U shape.

From Table 6 and Figure 12b, it can be seen that, compared with the classic U-shaped bending, the water-drop-shaped folding could not only reduce the maximum stress of the whole structure but also the core layers. There are two reasons for this. Firstly, according to the micro-element stress state of the material mechanics, the main stress distribution of the U-shaped normal bending stress is in the bending semicircle, while the water-drop-shaped design increases the length of the spline curve by *L* which is two times compared to the U-shape. Thus, the main stress distribution after the bending is not limited to the semicircular area which could reduce the stress concentration. Secondly, because there is no direct control of the droplet forming a semicircle, the radius of the rounded part is slightly larger than the initial design *R*. For the bending radius *R* = 2 mm, the rounded droplet is approximately equal to 2.286 mm. Thus, the stress is reduced by the inverse relationship between the radius of curvature and the normal bending stress. On the other hand, the reason why the stress reduction effect is not obvious for the large radius is that the final state water-droplet circle is close to the U-shaped circle. Therefore, for OLED flexible screen equipment which continues to pursue a small bending radius, water-drop-shaped folding is preferred because it can not only save space but also reduce the risk of structure failure.

### 4.4. Experimental Results

In previous research, Saleh et al. [32] systematically described the various bending methods of flexible electronic devices and the corresponding bending test devices, which has a certain significance for the design and selection of experiments.

Currently, it is difficult to detect the internal stacked structure stress. Instead, the maximum displacement distance of the flexible screen was measured to verify the accuracy of the model, because the three basic variables in the finite element can be expressed based on the nodal displacement matrix. Therefore, the finite element simulation results can be verified by the maximum slip distance during bending.

**Figure 13:**
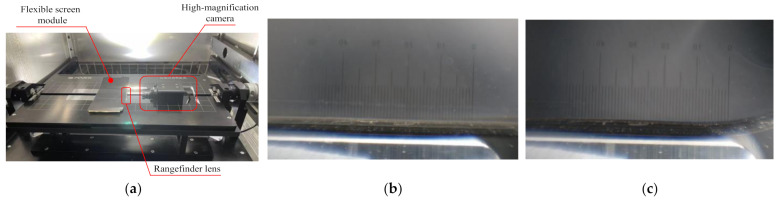
(**a**) Experimental schematic; (**b**) ranging before bending (units shown in μm); (**c**) ranging after bending (units shown in μm).

Before the experiment, the corresponding film was marked by laser engraving and other methods on the flexible screen module section. When the flexible screen module was bent, a CCD camera with a high-magnification telephoto lens was employed to continuously take photographs to obtain a complete record of the film layer dislocation. Due to the optical imaging function, a micro-range lens with an accuracy of 0.1 mm was placed on the side of the flexible screen. Adobe Illustrator were used to obtain the frame numbers at the beginning and end of bending, and the images were overlapped to measure the slip distance of the designated mark. The experimental equipment and result are shown in Figure 13. The maximum sliding distance of bending was recorded after the bending experiments, as shown in Table 7. In this experiment, the bending radius of the fixture board was set to 3 mm.

In order to reduce the influence of reading error during ranging, the mathematical expectation was calculated. Compared with the simulated ranging under the same bending radius, the difference ratio was less than 1%, which confirms the accuracy of the finite element model and the experimental results.

## 5. Conclusions and Suggestion

In this paper, finite element analysis of the bending model for the flexible screen was performed. For the common U-shaped bending, the maximum Mises stress increases rapidly as the bending radius decreases. The stack sequence of the OLED and BP layers, the most dangerous single layer and the profiles of the OCA layer were optimized. The optimization suggests the redistribution of the tensile zone and the compression zone are the key to the layer material selection. In order to validate the analysis, an imaging experiment was conducted to measure the maximum slip distance during bending. Further, a recently popular water-drop-shaped bending model was also discussed. The analysis implies that the bending would not only perform well with a small bending radius but also reduce the structure failure risk.

## Figures and Tables

**Table 1:** Sequence of stacking.

Number	1	2	3	4	5	6	7	8	9	10	11	12	13	14
Layer name	PD	OCA	TW	OCA	POL	OCA	OLED	OCA	BP	OCA	CPI	OCA	BMS	Glue
Thickness/μm	55	25	62	50	31	15	38	25	25	25	100	25	150	100
Mesh	6	4	5	5	4	4	5	4	4	4	6	4	6	8

**Table 2:** OCA parameters of forth-order Ogden equation.

i	$\mu_{i}/\mathrm{MPa}$	$\alpha_{i}$
1	−1.721 × 10^−2^	7.8758
2	6.675 × 10^−3^	2.5374
3	2.826 × 10^−2^	−0.4974
4	3.443 × 10^−2^	−15.7517

**Table 3:** OCA parameters of the second-order Prony equation.

i	$g_{i}$	$\tau_{i}$
1	0.29652	15.445
2	0.25903	124.29

**Table 4:** Plasticity parameters.

Yield Stress/MPa	Plastic Strain Increment
65	0
90	0.02
231	0.7

**Table 5:** Material parameters of each film.

Layer Name	Elastic Modulus/MPa	Poisson Ratio
PD	4000	0.33
TW	5600	0.33
POL	3769	0.33
OLED	16,355.43	0.3
BP	3500	0.33
CPI	2500	0.34
BMS	6750	0.275

**Table 6:** Maximum stress increment ratio of the core layer.

Core Layer		OLED	CPI	BMS
Incremental ratio	R2	17.993%	8.892%	14.671%
	R2.5	13.397%	16.879%	10.883%
	R3	5.798%	13.922%	5.453%

**Table 7:** Comparison of ranging results.

Experimental Ranging/mm	Mathematical Expectation/mm	Simulation Ranging/mm	Difference Ratio
0.7	0.698	0.694418	0.513%
0.698			
0.695			
0.7			
0.697			

## Data Availability

Not applicable.
